# Genome Comparison Reveals Mutation Hotspots in the Chloroplast Genome and Phylogenetic Relationships of* Ormosia* Species

**DOI:** 10.1155/2019/7265030

**Published:** 2019-08-21

**Authors:** Hongshan Liu, Zhihai Su, Shuiqing Yu, Jialin Liu, Xiaojuan Yin, Guowei Zhang, Wei Liu, Bin Li

**Affiliations:** ^1^State Key Laboratory of Tree Breeding and Forest Genetics, Key Laboratory of Tree Breeding and Cultivation of State Forestry Administration, Research Institute of Forestry, Chinese Academy of Forestry, Beijing 100091, China; ^2^Administration Bureau of Hongyashan State Owned Forest Farm of Hebei Province, Yixian 074200, China

## Abstract

The papilionoid legume genus* Ormosia *comprises approximately 130 species, which are distributed mostly in the Neotropics, with some species in eastern Asia and northeastern Australia. The taxonomy and evolutionary history remain unclear due to the lack of a robust species-level phylogeny. Chloroplast genomes can provide important information for phylogenetic and population genetic studies. In this study, we determined the complete chloroplast genome sequences of five* Ormosia* species by Illumina sequencing. The* Ormosia* chloroplast genomes displayed the typical quadripartite structure of angiosperms, which consisted of a pair of inverted regions separated by a large single-copy region and a small single-copy region. The location and distribution of repeat sequences and microsatellites were determined. Comparative analyses highlighted a wide spectrum of variation, with* trnK-rbcL*,* atpE-trnS-rps4*,* trnC-petN*,* trnS-psbZ-trnG*,* trnP-psaJ-rpl33,* and* clpP* intron being the most variable regions. Phylogenetic analysis revealed that* Ormosia *is in the Papilionoideae clade and is sister to the* Lupinus *clade. Overall, this study, which provides* Ormosia* chloroplast genomic resources and a comparative analysis of* Ormosia* chloroplast genomes, will be beneficial for the evolutionary study and phylogenetic reconstruction of the genus* Ormosia* and molecular barcoding in population genetics and will provide insight into the chloroplast genome evolution of legumes.

## 1. Introduction

The genus* Ormosia* Jacks. (Fabaceae, Papilionoideae) comprises approximately 130 species and has a disjunct distribution between the Neotropics and the eastern Asian and northeastern Australian Tropics, i.e., from southern India and southern China to northeastern Australia [1, 2].* Ormosia* was defined as a segregate genus by the following combination of morphological characters: flowers with distinct, imbricate calyx lobes; an incurved style with a terminal or lateral (usually bilobed) stigma; and predominantly red, black, or bicolored seeds with a hard testa. Molecular phylogenetic analyses based on the chloroplast* matK* and* trnL* intron sequences in large-scale phylogenetic studies of papilionoid genera have consistently recovered a monophyletic* Ormosia*, yet sampling within the genus has been limited [1, 3].* Ormosia *diverged as an early branch within the Genistoid clade, which is one of the fundamental lineages of papilionoid legumes [3]. The results also showed that* matK* and* trnL* intron sequences lacked variations among the species. However, only a few genomic resources have been explored in this genus. In GenBank, there are presently fewer than 500 sequences of* Ormosia* species.

In recent years, the chloroplast genome resources have been widely used in plant systematics and species identification [4–8]. The chloroplast genome is inherited in a maternal manner in the majority of plants and is smaller in size and has very low recombination compared with that of the nuclear and mitochondria genome [9]. Moreover, the chloroplast genome has a moderate rate of nucleotide evolution, which makes the chloroplast genome suitable for species identification and for phylogenetic studies at different taxonomic levels [10].

Most of the chloroplast genomes in angiosperms have a typical quadripartite structure, with two copies of inverted repeats (IRs) separating the large single-copy (LSC) and small single-copy regions (SSC) and the genome size ranging from 120 to 170 kb in length. A comparative analysis of the complete chloroplast genomes played an important role in understanding the chloroplast genome evolution. In this paper, we investigated the complete chloroplast genomes of five* Ormosia* species through next-generation sequencing (NGS). The objectives of this study were (i) to describe the structure of the* Ormosia* chloroplast genome; (ii) to identify highly divergent regions in the* Ormosia* chloroplast genome which suit DNA barcodes; and (iii) to calibrate the phylogenetic position of* Ormosia* based on phylogenomic analysis.

## 2. Materials and Methods

### 2.1. Plant Materials and DNA Extraction

The fresh and healthy leaves of five* Ormosia* species were collected from the Subtropical Forestry Experimental Center of Chinese Academy of forestry, Fenyi, Jiangxi (*O. henryi *and* O. hosiei*), Jiangmen, Guangdong (*O. emarginata*), Bawangling National Nature Reserve, Hainan (*O. xylocarpa*), and Longmen, Huizhou, Guangdong (*O. semicastrata*). Voucher specimens were deposited in the herbaria of the Institute of Botany (PE), China Academy of Sciences. Fresh leaves from each accession were immediately dried with silica gel prior to DNA extraction. The total genomic DNA was extracted following the method of Li et al. [11] and was purified using the Wizard DNA Clean-Up System (Promega, Madison, WI, USA). The DNA quality was assessed based on spectrophotometry and electrophoresis in 1% (w/v) agarose gel.

### 2.2. Illumina Sequencing, Assembly, and Annotation

The DNA was sheared to fragments of 400~600 bp using an ultrasonicator. Paired-end libraries were prepared with the NEBNext® Ultra™ DNA Library Prep Kit. The genome was then sequenced using the HiSeq X Ten platform (Illumina, Santiago, CA, USA).

The paired-end reads were qualitatively assessed and assembled with SPAdes 3.6.1 [12]. Chloroplast genome sequence contigs were selected from SPAdes software by performing a BLAST search using the* Lupinus albus* chloroplast genome sequence as a reference (GenBank accession number: KJ468099) and then were assembled with Sequencher 5.4.5 (Gene Codes, Ann Arbor, MI). To verify the assembly, four boundaries between the single-copy (SC) and the inverted repeat (IR) regions of the assembled sequences were confirmed by PCR amplification and Sanger sequencing using the primers by Dong et al. [13]. Chloroplast genome annotation was performed with Plann [14] using the* Lupinus albus *as reference sequence from GenBank. A chloroplast genome map was drawn using Genome Vx software [15].

### 2.3. Analysis of Tandem Repeats and Single Sequence Repeats

Five types of repeat sequences, including forward repeat, reverse repeat, complement repeat, palindromic repeat, and tandem repeat, were identified in the* Ormosia *chloroplast genomes. We used REPuter to identify forward repeat, reverse repeat, complement repeat, and palindromic repeat [16], in which the similarity percentage of the two repeat copies was at least 90%, the minimum repeat size was 30 bp, and the hamming distance was 3. Tandem repeats were identified using the web-based Tandem Repeats Finder (https://tandem.bu.edu/trf/trf.html), with 2, 7, and 7 set for the alignment parameters match, mismatch, and indel, respectively.

Single sequence repeats (SSRs) were identified by GMAT [17] with the parameters set at >10 for mononucleotide, >5 for dinucleotide, >4 for trinucleotide, and >3 for tetranucleotide, pentanucleotide, and hexanucleotide SSRs.

### 2.4. Comparison of Whole Chloroplast Genomes and Divergent Hotspot Identification

The mVISTA program (http://genome.lbl.gov/vista/mvista/submit.shtml) with Shuffle-LAGAN mode [18] was used to compare the* Ormosia* chloroplast genomes. The* O. henryi *chloroplast genome was used as a reference.

All five* Ormosia* sequenced chloroplast genomes were aligned using MAFFT v7 [19], assuming collinear genomes for the full alignment, and then were adjusted manually using Se-Al 2.0 [20]. A sliding window analysis was conducted to generate the nucleotide diversity of the chloroplast genome using the DnaSP v5.10 software [21]. The step size was set to 100 bp, with an 800-bp window length.

### 2.5. Phylogenetic Reconstruction

Eighty-one protein-coding sequences were present in 70 species from the family Fabaceae and one species from Moraceae as an outgroup were used for the phylogenetic reconstruction. The chloroplast genomes of these species were downloaded from GenBank (Table S1). Gene alignment was performed using MAFFT v7 [19]. Phylogenetic trees were constructed by the maximum likelihood (ML) and Bayesian inference (BI) analyses methods.

The program ModelFinder was used to find the optimal substitution mode [22], using both the Bayesian information criterion and the Akaike information criterion. Maximum likelihood (ML) analyses were performed using RAxML v.8.1.24. Statistical support for the branches (BS) was calculated by rapid bootstrap analyses with 1000 replicates.

Bayesian inference was conducted using MrBayes v3.2.2 [23] using the GTR+G+I model on the CIPRES Science Gateway. The default priors were utilized, along with the default heating scheme (one cold and three heated chains), and runs were conducted for 10 million generations with trees sampled every 1000 generations. The first 25% percent of trees from all runs were discarded as burn-in.

## 3. Results

### 3.1. Genome Sequencing and Assembly

Using the Illumina HiSeq X Ten system, the total DNA from five species of* Ormosia* was sequenced to produce 165,518,310–342,489,92 paired-end raw reads (150 bp average read length) per species. After screening, these paired-end reads through alignment with themselves, 209,546 to 813,022 chloroplast genome reads were extracted with 181 X to 702 X coverage (Table 1). The accuracy of inverted repeat junction regions in assembled sequences was further confirmed by PCR amplification and Sanger sequencing with specific primers. The finished, high-quality* Ormosia* chloroplast genome sequences that were thus obtained were used in the following analyses and were submitted to GenBank (accession numbers, MH571753, MH571754, and MK105448- MK105450).

### 3.2. Chloroplast Genomes Features of* Ormosia* Species

**Figure 1:**
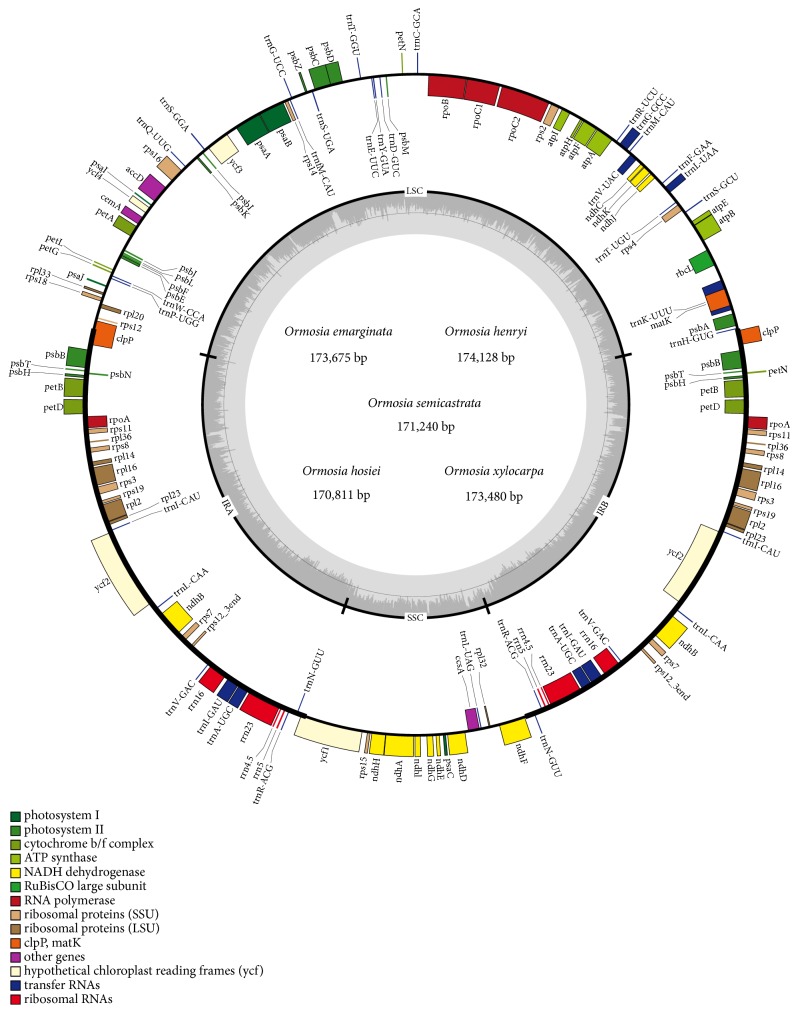
*Gene map of Ormosia chloroplast genome*. The genes inside and outside the circle are transcribed in the clockwise and counterclockwise directions, respectively. Genes in different functional groups are shown in different colors. The thick lines indicate the extent of the inverted repeats (IRa and IRb) that separate the genomes into small single-copy (SSC) and large single-copy (LSC) regions.

The five* Ormosia* chloroplast genomes ranged from 170,811 to 174,128 base pairs in length, with* Ormosia henryi *being the largest and* Ormosia hosiei *the smallest. All chloroplast genomes shared the common feature comprising two copies of IR (40,034–40,633 bp) separated by the LSC (71,728–74,231 bp) and SSC (18,295– 18,798 bp) regions (Figure 1, Table 2). The overall GC content was 35.7-36.0%, which indicated nearly identical levels among the five complete* Ormosia* chloroplast genomes.

*Ormosia* chloroplast genomes all have 112 different genes arranged in the same order, including 78 protein-coding genes, 30 tRNAs, and 4 rRNAs. Among these genes, twelve of the protein-coding genes and six of the tRNA genes contained introns; 16 genes harbored a single intron and two genes (*ycf3* and* clpP*) harbored two introns. 42 genes were duplicated in the IR region, including 31 protein-coding genes, 7 tRNA genes, and 4 rRNA genes (*rrn5, rrn4.5, rrn23, *and* rrn16*). The* trnK-UUU* had the largest intron, which contained the* matK* gene. The 5′-end exon of the* rps12* gene was located in the LSC region, and the intron and two copies of 3′-end exon were located in the IR regions.

### 3.3. IR Expansion and Contraction

**Figure 2:**
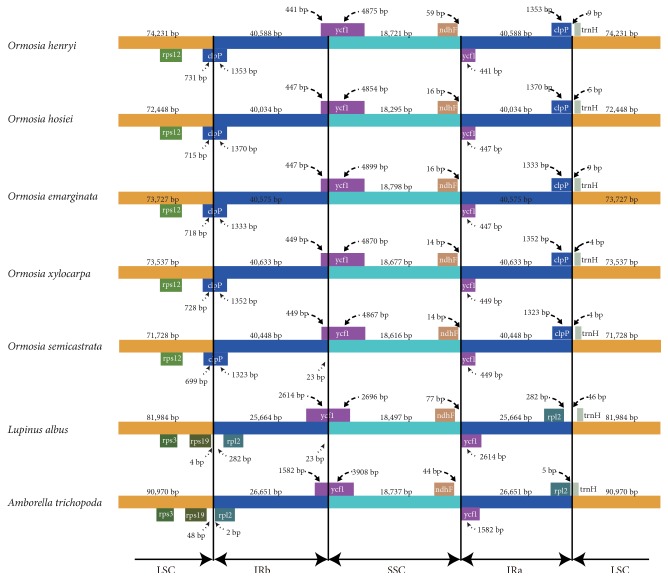
Comparison of the border positions of the LSC, SSC, and IR regions.

The IR boundary regions of five* Ormosia* species and* Lupinus albus *(Fabaceae) and* Amborella trichopoda* were compared, and the results showed that the border of the* Ormosia* chloroplast genomes was slightly different from that of other genomes (Figure 2). In* Ormosia*, the boundary of IRb/LSC occurred within the gene* clpP*, resulting in the duplication of a portion of this gene (1,323-1,370 bp) in the IR region. The boundary of IRb/LSC in* Lupinus albus* and* Amborella trichopoda* occurred between* rps19 *and* rpl2 *and between* rpl2 *and* trnH-GUG *on the IRa/LSC side, with 0 and 282 noncoding nucleotides between these two genes. The IRa/SSC border extended into* ycf1*, resulting in a pseudogene in the five* Ormosia* species. The length of the* ycf1 *pseudogene was 4,854-4,899 bp in* Ormosia*, 2,696 bp in* Lupinus albus*, and 3,908 bp in* Amborella trichopoda*. Furthermore,* ndhF *deviated from the IRb/SSC in* Ormosia *by 14-59 bp. There were 4-9 bp of noncoding sequence between IRa/LSC border and the 3'-end of gene* trnH-GUG* in the LSC region. Taken together, the IR in* Ormosia* had a 15 kb expansion compared with other lineages, and the IR boundary regions varied slightly within the* Ormosia* chloroplast genomes.

### 3.4. Analysis of Repeat Elements

**Figure 3:**
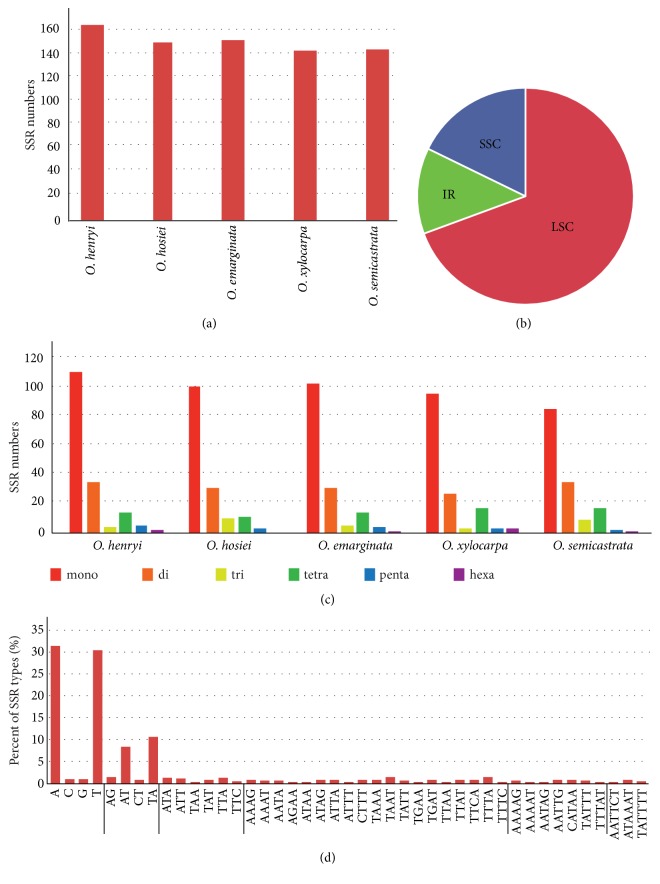
*Analysis of perfect simple sequence repeats (SSRs) in five Ormosia chloroplast genomes*. (a) Number of SSRs detected in five chloroplast genomes. (b) Frequency of identified SSRs in LSC, IR, and SSC regions. (c) Number of SSR types detected in five chloroplast genomes. (d) Frequency of identified SSR motifs in different repeat class types.

Each* Ormosia* chloroplast genomes contained 147 to 169 SSRs (Figure 3(a)). Among these SSRs, most were located in the LSC/SSC regions (85.2-88.1%, Figure 3(b)). The average of mono-, di-, tri-, and tetranucleotide SSRs were 62.92%, 20.54%, 4.01%, and 9.43%, respectively. Hexanucleotide SSRs were very rare across the chloroplast genomes (Figure 3(c)). SSRs in* Ormosia* chloroplast genomes were especially rich in AT and rarely contained CG (Figure 3(d)). Almost all SSRs (61.37%) were mononucleotide A/T repeats; C/G mononucleotide SSRs were rarely present (1.55%). AT/TA repeats were the most common (90.57%) among dinucleotide SSRs.

**Figure 4:**
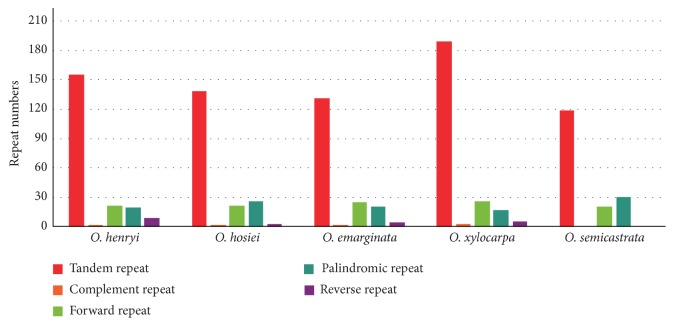
Analysis of repeated sequences in five* Ormosia* chloroplast genomes.

In addition to the SSRs, we employed REPuter and the Tandem Repeats Finder to analyze the repeat sequences of the five* Ormosia *chloroplast genomes (Figure 4). We classified sequence repeat motifs into five categories: forward, reverse, complement, palindromic, and tandem repeats.* Ormosia *contained 21-26 forward repeats, 0-9 reverse repeats, 0-2 complement repeats, 17-30 palindromic repeats, and 118-188 tandem repeats.

### 3.5. Sequence Divergence and Divergence Hotspot Regions

**Figure 5:**
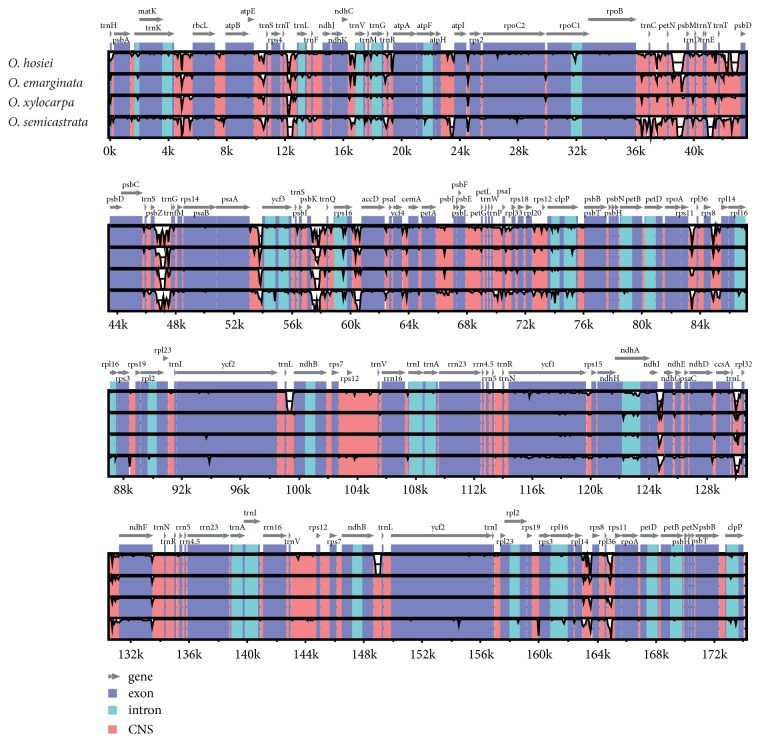
*Sliding window analysis of the Ormosia chloroplast genomes (window length: 800 bp; step size: 100 bp)*.* x*-axis: position of the midpoint of a window;* y*-axis: nucleotide diversity of each window.

A comparative analysis based on mVISTA was performed among the five chloroplast genomes of* Ormosia* to investigate the levels of sequence divergence (Figure 5). VISTA-based similarity graphical information portrays sequence identity among the five* Ormosia* chloroplast genomes with a reference to the* O. henryi *chloroplast genomes. The organization of the chloroplast genome among* Ormosia* was essentially colinear and gene order conservation. The results also showed that the IR regions and coding region were more conserved than SC region and noncoding regions.

**Figure 6:**
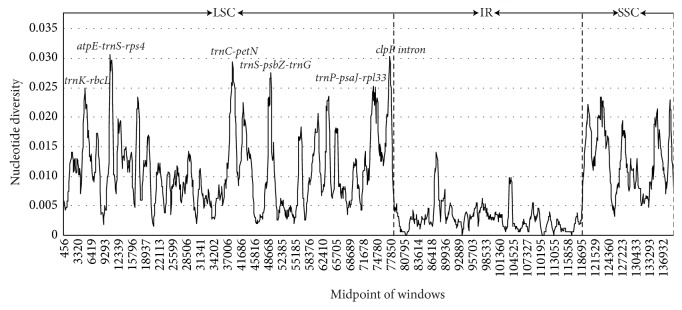
*Visualization of genome alignment of the chloroplast genomes of five Ormosia species using O. henryi as reference using mVISTA*. The* y*-scale axis represents the percent identity within 50%–100%. Dashed rectangles indicate highly divergent regions among* Ormosia*.

To identify the sequence divergence hotspots, the nucleotide diversity (pi, *π*) value within 800 bp was calculated (Figure 6) with DnaSP 5.0 software. In the* Ormosia* chloroplast genomes, the pi values varied from 0 to 0.03063. The IR region was more conserved than that of the LSC and SSC regions among the five genomes. Six hypervariable regions (Pi > 0.025) were uncovered among the* Ormosia* chloroplast genomes. They were* trnK-rbcL*,* atpE-trnS-rps4*,* trnC-petN*,* trnS-psbZ-trnG*,* trnP-psaJ-rpl33,* and* clpP* intron. All six regions were located in the LSC region.

### 3.6. Phylogenomic Analysis

Chloroplast phylogenomics has been proved to be effective in resolving complex relationships at the order level, such as Saxifragales [6]; family level, such as Nelumbonaceae [5]; and the lower taxonomic level, such as* Juglans* [4] and* Forsythieae* [8]. In this study, we used 81 protein-coding genes to calibrate the phylogenetic position of* Ormosia* in the Fabaceae.

**Figure 7:**
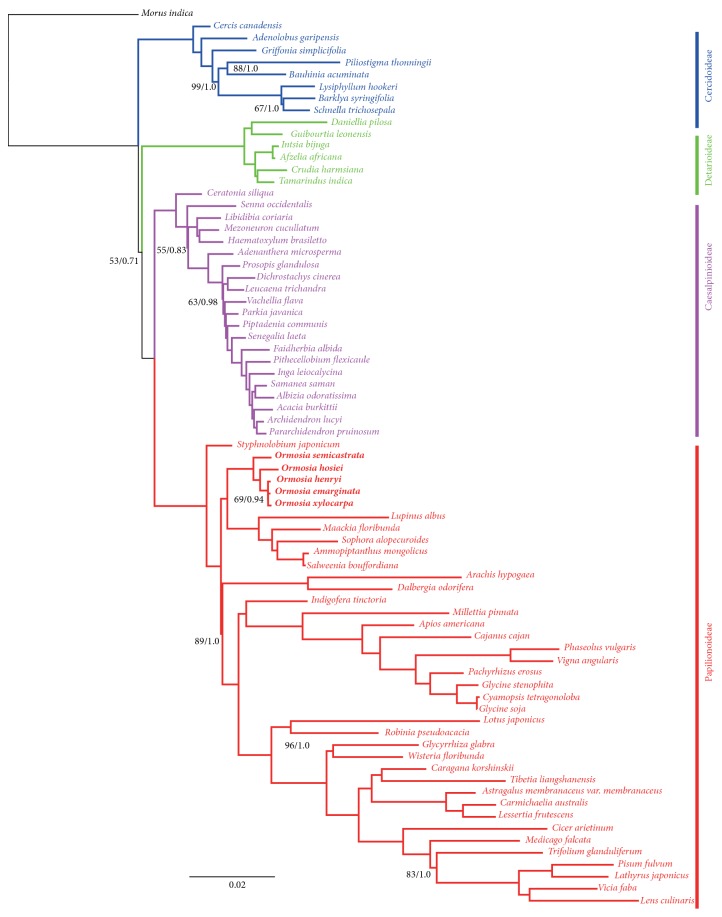
*Phylogenetic tree obtained from maximum likelihood and Bayesian inference methods of 81 genes for 82 taxa*. Numbers above nodes indicate ML bootstrap support value (ML-BP)/Bayesian posterior probability (BI-PP). Nodes with 100 ML-BP/1.0 BI-PP/100 MP-BP are not marked.

ML and Bayesian analyses based on 81 protein-coding genes produced identical tree topologies, with 100% bootstrap support (BS) form ML and 1.0 Bayesian posterior probabilities (PP) at nearly every node (Figure 7, Figure S1). The phylogeny was congruent with the published* matK* gene-based phylogenies and showed Cercidoideae as a basal, Caesalpinioideae and Papilionoideae forming sister groups [1]. The result showed that Dialioideae was sister to Caesalpinioideae + Papilionoideae, though with lower bootstrap support and posterior probability values (53 BS/0.71 PP). There were two reasons that might explain the tree topology with lower bootstrap support. Firstly, the inferred phylogenetic trees combine short and long internodes branches, indicating rapid radiation [6, 24]. Secondly, incomplete lineage sorting is proposed as a potential explanation for incongruence among characters [25, 26].* Ormosia *was in the Papilionoideae clade and was sister to the* Lupinus *clade.

Phylogenetic analysis based on 81 protein-coding genes (Figure 7) successfully resolved relationships among the sampled species of* Ormosia. O. semicastrata* occupied the most basal position, which was sister to the rest of the* Ormosia* species.* O. hosiei* was sister to* O. henryi, O. emarginata,* and* O. xylocarpa*, which formed a clade.

## 4. Discussion

### 4.1. Chloroplast Genome Evolution of* Ormosia*

In this study, using the next-generation sequencing method, we sequenced five new chloroplast genomes of* Ormosia*. The complete chloroplast genomes ranged from 170,811 to 174,128 bp, which is longer compared to that of the other angiosperms. The chloroplast genomes of* Ormosia* species were structurally conserved, and no rearrangement events were detected in this study. Meanwhile, the genome divergence was low. mVISTA results revealed high similarities among chloroplast genomes, which suggested that the* Ormosia *cpDNAs were rather conserved. The* Ormosia* chloroplast genome was structurally similar to that of most angiosperms chloroplast genomes and the IR region showed lower sequence divergence than SSC and LSC regions possibly due to copy corrections between the IR sequences by gene conversion [27].

The organization of the* Ormosia* chloroplast genomes was similar to that of the angiosperm genome, except for the IR expansion. The boundaries of repeat/single copy represent highly variable regions and often influence the genome size of the chloroplast genome. The information of the IR expansion and contraction can be used to study the genome evolution among plant lineages. In this study, by comparing the inverted repeat/single-copy (IR/SC) boundaries, we detected a 15 kb IR expansion in* Ormosia*. The position of all four IR/SC junctions can vary even among closely related species in angiosperm chloroplast genomes. The shifts are small among the* Ormosia *species, involving up to several hundred bp (Figure 2). Larger IR expansions occur less frequently and outnumber large contractions [28]. For example, there is a 10 kb IR contraction in Schisandraceae [29]. In* Petroselinum*, the IR contracted ~1.5 kb at the IRB-LSC boundary compared with other Apiales species [30].

The IRb and LSC boundaries typically occur between* rpl2* and* trnH-GUG* in most angiosperms [31]. Several elegant models have been proposed to explain the diversification of the IR boundary regions sequences. Goulding et al. [32] and Wang et al. [31] proposed a model that the double-strand break in the IR and LSC boundaries followed by strand invasion and recombination to explain the larger IR expansion. The second model is the recombination between the short repeats or poly(A) of tRNA genes, which may affect the position of the IR boundary [30]. The third model is the indels, which caused a mismatch that resulted in the upstream sequence becoming a single copy [33].

Variation in the chloroplast genome size and gene order within groups is relatively rare. However, the Fabaceae chloroplast genome exhibited significant size variation, chloroplast genome rearrangements, and gene and intron losses [28, 34]. There were also many IR boundary shifts in the legumes [28, 35]. Therefore, further research with expanded sampling is urgently needed to determine the IR boundary shifts and genome rearrangements in the Fabaceae.

### 4.2. Highly Variable Chloroplast Markers for Evaluating* Ormosia *Phylogeny and DNA Barcoding

Because of the more than 120 species, great morphological diversity, disjunct distribution of the genus* Ormosia*, its DNA barcoding and species phylogenetic relationships are still difficult to unravel. Only a few studies focused on the phylogeny and taxonomy of* Ormosia *by molecular phylogeny. The chloroplast genome markers, the* rbcL*,* matK*,* trnH-psbA*,* trnL-F*,* ndhF*,* rpoB*, and* ycf1* genes, have been used widely to investigate taxonomy and DNA barcoding [10, 36, 37]. Nevertheless, increasingly more studies showed that those markers had low discriminatory power and insufficient information for phylogenetic analysis [38, 39].

The indel and single nucleotide substitute mutation events were not random but were clustered as “hotspots” in the chloroplast genome. Those highly variable regions that evolve very rapidly and meet the criteria required to be a DNA barcode. The strategy of searching the potential DNA barcodes has been successfully applied to* Diospyros* [40], Yam [41],* Oryza *[42], and* Lagerstroemia* [43]. Based on the five compared* Ormosia* chloroplast genomes, six highly variable regions (*trnK-rbcL*,* atpE-trnS-rps4*,* trnC-petN*,* trnS-psbZ-trnG*,* trnP-psaJ-rpl33,* and* clpP* intron) were identified. The regions* trnS-psbZ-trnG *and* clpP* intron have been the focus of previous studies to assess the DNA barcodes in angiosperms [10]. Therefore, further work on investigating whether these markers could be recommended as effective, specific barcodes for* Ormosia* species is necessary.

## Figures and Tables

**Table 1:** Summary of the sequencing data for five *Ormosia species*.

Species	Locality	Voucher	Raw data no.	Mapped reads no.	Mapped to reference genome (%)	Chloroplast genome coverage (X)
*O. henryi *	Fenyi, Jiangxi	BOP214710	34,248,992	390,755	1.14%	337
*O. hosiei*	Fenyi, Jiangxi	BOP214711	39,701,622	466,122	1.17%	409
*O. emarginata*	Jiangmen, Guangdong	BOP216254	16,518,310	813,022	4.92%	702
*O. xylocarpa*	Bawangling, Hainan	BOP216381	23,924,424	209,546	0.88%	181
*O. semicastrata *	Longmen, Guangdong	BOP217157	23,652,340	219,828	0.93%	193

**Table 2:** Summary statistics for assembly of five *Ormosia* species chloroplast genomes.

Species	*O. henryi*	*O. hosiei*	*O. emarginata*	*O. xylocarpa*	*O. semicastrata*
Length (bp)	174,128	170,811	173,675	173,480	171,240
LSC (bp)	74,231	72,448	73,727	73,537	71,728
IR (bp)	40,588	40,034	40,575	40,633	40,448
SSC (bp)	18,721	18,295	18,798	18,677	18,616
Gene number	110	110	110	110	110
Protein coding genes	76	76	76	76	76
tRNA	30	30	30	30	30
rRNA	4	4	4	4	4
GC content (%)	35.7	36	35.8	35.9	35.9
Accession number	MH571754	MH571753	MK105448	MK105449	MK105450

## Data Availability

The five* Ormosia* chloroplast genomes are available in GenBank database (accession numbers: MH571753, MH571754, and MK105448- MK105450).

## Supplementary Materials

Supplementary Materials Table S1. A list of the 72 taxa sampled from GenBank in this study for phylogeny analysis. Figure S1. Phylogenetic tree reconstruction of 82 taxa using Bayesian inference methods based on 81 genes chloroplast genome sequences.
