# Supplementary material for: Genome Comparison Reveals Mutation Hotspots in the Chloroplast Genome and Phylogenetic Relationships of Ormosia Species
Source: Biomed Res Int. 2019 Aug 21;2019:7265030. doi: 10.1155/2019/7265030 (PMC6720362; doi:10.1155/2019/7265030)

Genome Comparison Reveals Sequence Divergence and Mutation Hotspots in the Chloroplast Genome of *Ormosia* Species

Hongshan Liu[1,2†], Zhihai Su[2†], Shuiqing Yu[2], Jialin Liu[2], Xiaojuan Yin[2], Guowei Zhang[2], Wei Liu[2], Bin Li[1*]

[1]State Key Laboratory of Tree Breeding and Forest Genetics, Key Laboratory of Tree Breeding and Cultivation of State Forestry Administration, Research Institute of Forestry, Chinese Academy of Forestry, Beijing, 100091, China;

[2]Administration Bureau of Hongyashan State Owned Forest Farm of Hebei Province， Yixian, 074200, China

† Hongshan Liu and Zhihai Su have contributed equally to this work.

Correspondence: Bin Li, E-mail: libin1200@163.com

Table S1. A list of the 72 taxa sampled from GenBank in this study for phylogeny analysis.

Figure S1. Phylogenetic tree reconstruction of 82 taxa using Bayesian inference methods based on 81 genes chloroplast genome sequences.

Table S1. A list of the 72 taxa sampled from GenBank in this study for phylogeny analysis.

Organism	Accession	Sequence Length	%GC	Family	Subfamily
Libidibia coriaria	KJ468095	158045	36.50%	Fabaceae	Caesalpinioideae
Mezoneuron cucullatum	KU569489	158357	36.40%	Fabaceae	Caesalpinioideae
Haematoxylum brasiletto	KJ468097	157728	36.70%	Fabaceae	Caesalpinioideae
Senna occidentalis	MF358692	159993	36.20%	Fabaceae	Caesalpinioideae
Acacia burkittii	LN885253	174711	36.10%	Fabaceae	Caesalpinioideae
Senegalia laeta	KY100269	162754	35.80%	Fabaceae	Caesalpinioideae
Vachellia flava	KY100263	165829	35.30%	Fabaceae	Caesalpinioideae
Albizia odoratissima	KX852437	174861	35.60%	Fabaceae	Caesalpinioideae
Archidendron lucyi	KX852438	176870	35.20%	Fabaceae	Caesalpinioideae
Faidherbia albida	KX852440	175646	35.30%	Fabaceae	Caesalpinioideae
Inga leiocalycina	KT428296	175489	35.50%	Fabaceae	Caesalpinioideae
Pararchidendron pruinosum	KX852441	176692	35.30%	Fabaceae	Caesalpinioideae
Pithecellobium flexicaule	KX852444	178887	35.10%	Fabaceae	Caesalpinioideae
Samanea saman	KX852445	176717	35.30%	Fabaceae	Caesalpinioideae
Adenanthera microsperma	KX852436	159389	36.50%	Fabaceae	Caesalpinioideae
Dichrostachys cinerea	KX852439	161240	35.90%	Fabaceae	Caesalpinioideae
Leucaena trichandra	KT428297	164692	35.60%	Fabaceae	Caesalpinioideae
Parkia javanica	KX852442	161681	35.90%	Fabaceae	Caesalpinioideae
Piptadenia communis	KX852443	162552	35.90%	Fabaceae	Caesalpinioideae
Prosopis glandulosa	KJ468101	163040	35.90%	Fabaceae	Caesalpinioideae
Ceratonia siliqua	KJ468096	156367	36.70%	Fabaceae	Caesalpinioideae
Adenolobus garipensis	KY806280	151705	36.90%	Fabaceae	Cercidoideae
Barklya syringifolia	MF135594	158740	36.40%	Fabaceae	Cercidoideae
Bauhinia acuminata	MF135595	155548	36.50%	Fabaceae	Cercidoideae
Lysiphyllum hookeri	MF135601	154055	36.60%	Fabaceae	Cercidoideae
Piliostigma thonningii	MF135598	165416	36.40%	Fabaceae	Cercidoideae
Schnella trichosepala	MF135599	159722	36.20%	Fabaceae	Cercidoideae
Griffonia simplicifolia	MF135596	157909	36.30%	Fabaceae	Cercidoideae

Cercis canadensis	KF856619	158995	36.20%	Fabaceae	Cercidoideae
Afzelia africana	KX673213	159603	36.10%	Fabaceae	Detarioideae
Intsia bijuga	KX673214	159215	36.20%	Fabaceae	Detarioideae
Daniellia pilosa	MG599083	159243	36.00%	Fabaceae	Detarioideae
Crudia harmsiana	MG599082	158253	36.40%	Fabaceae	Detarioideae
Guibourtia leonensis	MG564755	159295	36.10%	Fabaceae	Detarioideae
Tamarindus indica	KJ468103	159551	36.20%	Fabaceae	Detarioideae
Caragana korshinskii	KX289923	129331	34.40%	Fabaceae	Papilionoideae
Tibetia liangshanensis	MF193597	123372	34.70%	Fabaceae	Papilionoideae
Cicer arietinum	EU835853	125319	33.90%	Fabaceae	Papilionoideae
Arachis hypogaea	KJ468094	156395	36.40%	Fabaceae	Papilionoideae
Dalbergia odorifera	MF668133	156064	36.10%	Fabaceae	Papilionoideae
Lathyrus japonicus	KJ806194	124242	34.90%	Fabaceae	Papilionoideae
Lens culinaris	KF186232	123096	34.40%	Fabaceae	Papilionoideae
Pisum fulvum	MG458702	120837	34.90%	Fabaceae	Papilionoideae
Vicia faba	KF042344	123722	34.60%	Fabaceae	Papilionoideae
Astragalus membranaceus var. membranaceus	KX255662	123623	34.10%	Fabaceae	Papilionoideae
Carmichaelia australis	MF597719	122805	34.30%	Fabaceae	Papilionoideae
Glycyrrhiza glabra	KF201590	127943	34.20%	Fabaceae	Papilionoideae
Lessertia frutescens	MF286764	122700	34.20%	Fabaceae	Papilionoideae
Lupinus albus	KJ468099	154140	36.50%	Fabaceae	Papilionoideae
Cyamopsis tetragonoloba	MF352008	152530	35.40%	Fabaceae	Papilionoideae
Indigofera tinctoria	KJ468098	158367	35.80%	Fabaceae	Papilionoideae
Lotus japonicus	AP002983	150519	36.00%	Fabaceae	Papilionoideae
Millettia pinnata	JN673818	152968	34.80%	Fabaceae	Papilionoideae
Wisteria floribunda	KM103376	130960	34.30%	Fabaceae	Papilionoideae
Apios americana	KF856618	148772	35.50%	Fabaceae	Papilionoideae
Cajanus cajan	KU729879	152242	35.00%	Fabaceae	Papilionoideae
Glycine stenophita	KC893634	152618	35.30%	Fabaceae	Papilionoideae
Glycine soja	KF611800	152217	35.40%	Fabaceae	Papilionoideae
Pachyrhizus erosus	KJ468100	151947	35.30%	Fabaceae	Papilionoideae
Phaseolus vulgaris	DQ886273	150285	35.40%	Fabaceae	Papilionoideae
Vigna angularis	AP012598	151683	35.20%	Fabaceae	Papilionoideae
Robinia pseudoacacia	KJ468102	154835	35.90%	Fabaceae	Papilionoideae
Maackia floribunda	KX388160	154541	36.50%	Fabaceae	Papilionoideae

Salweenia bouffordiana	MF449303	153730	36.80%	Fabaceae	Papilionoideae
Sophora alopecuroides	MF100928	154108	36.60%	Fabaceae	Papilionoideae
Styphnolobium japonicum f. violaceum	KY872756	158837	36.10%	Fabaceae	Papilionoideae
Ammopiptanthus mongolicus	KY034453	153935	36.90%	Fabaceae	Papilionoideae
Medicago falcata	KX831887	124430	34.00%	Fabaceae	Papilionoideae
Trifolium glanduliferum	KJ788285	126149	34.50%	Fabaceae	Papilionoideae
Morus indica	DQ226511	158484	36.40%	Moraceae	

Figure S1. Phylogenetic tree reconstruction of 82 taxa using Bayesian inference methods based on 81 genes chloroplast genome sequences.

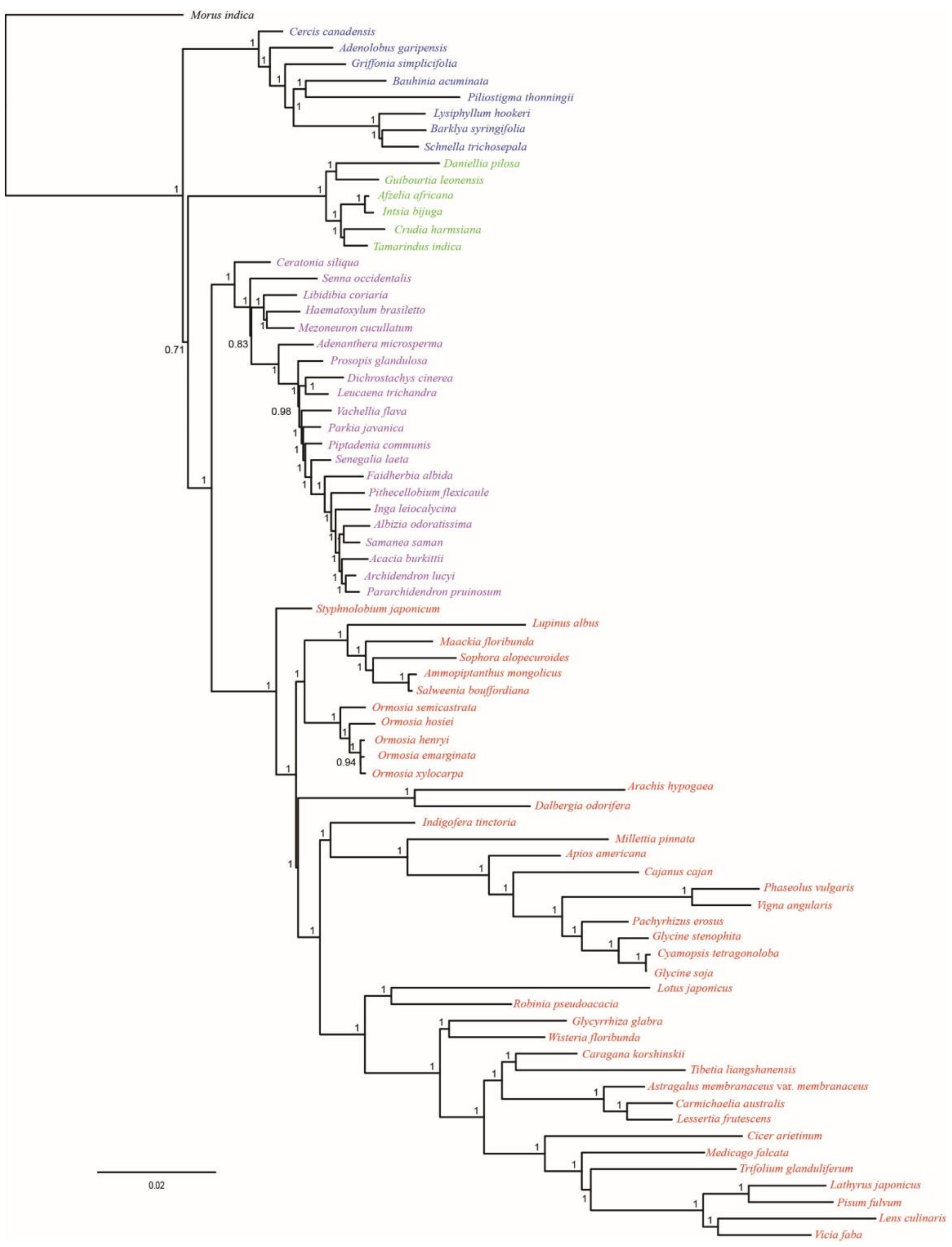

Supplement: Supplementary Materials — Table S1. A list of the 72 taxa sampled from GenBank in this study for phylogeny analysis. Figure S1. Phylogenetic tree reconstruction of 82 taxa using Bayesian inference methods based on 81 genes chloroplast genome sequences. [file 7265030.f1.pdf]
